# Vaccination dropout and wealth related inequality among children aged 12–35 months in remote and underserved settings of Ethiopia: a cross-sectional evaluation survey

**DOI:** 10.3389/fped.2023.1280746

**Published:** 2023-10-24

**Authors:** Fisseha Shiferie, Samson Gebremedhin, Gashaw Andargie, Dawit A. Tsegaye, Wondwossen A. Alemayehu, Legese Alemayehu Mekuria, Tamiru Wondie, Teferi Gedif Fenta

**Affiliations:** ^1^ Project HOPE Ethiopia Country Office, Addis Ababa, Ethiopia; ^2^ School of Pharmacy, Addis Ababa University, Addis Ababa, Ethiopia; ^3^ School of Public Health, Addis Ababa University, Addis Ababa, Ethiopia; ^4^ Project HOPE Headquarter, Washington, DC, United States

**Keywords:** vaccination, dropout rate, inequality, remote, underserved, Ethiopia

## Abstract

**Background:**

Vaccination is one of the most cost-effective public health interventions that prevents millions of deaths. Although immunization coverage is increasing globally, many children in low- and middle-income countries drop out of the vaccination continuum. This study aimed at determining vaccination dropout rates and predictors in children aged 12–35 months in remote and underserved areas of Ethiopia.

**Methods:**

This study was part of a cross-sectional evaluation survey that was conducted in 2022 in Ethiopia. The study settings include pastoralist, developing & newly established regions, conflict affected areas, urban slums, internally displaced populations and refugees. A sample of 3,646 children aged 12–35 months were selected using a cluster sampling approach. Vaccination dropout was estimated as the proportion of children who did not get the subsequent vaccine among those who received the first vaccine. A generalized estimating equation was used to assess determinants of the dropout rate and findings were presented using an adjusted odds ratio with 95% confidence interval. Concentration curve and index were used to estimate wealth related inequality of vaccination dropout.

**Results:**

A total of 3,646 caregivers of children participated in the study with a response rate of 97.7%. The BCG to Penta-3 (52.5%), BCG to MCV-2 (57.4%), and Penta-1 to Penta-3 (43.9%) dropouts were all high. The highest Penta-1 to Penta-3 dropout rate was found in developing regions (60.1%) and the lowest was in urban slums (11.2%). Caregivers who were working outside their homes [AOR (95% CI) = 3.67 (1.24–10.86)], who had no postnatal care follow-up visits [AOR (95%CI) = 1.66 (1.15–2.39)], who did not receive a service from a skilled birth attendant [AOR (95%CI) = 1.64 (1.21–2.27)], who were older than 45 years [AOR (95% CI) = 12.49 (3.87–40.33)], and who were less gender empowered [AOR (95%CI) = 1.63 (1.24–2.15)] had increased odds of Penta-1 to Penta-3 dropout. The odds of dropout for children from poor caregivers was nearly two times higher compared to their wealthy counterparts [AOR (95%CI) = 1.87 (1.38–2.52)].

**Conclusion:**

Vaccination dropout estimates were high among children residing in remote and underserved settings. Poor wealth quintile, advanced maternal age, low women empowerment, and limited utilization of maternity care services contributed to vaccination dropout.

## 1. Introduction

Vaccination is one of the most important and cost-effective public health interventions that prevents millions of deaths annually. Even though immunization coverage is increasing globally, many children in low- and middle-income countries drop out of the vaccination continuum (1–3). The global coverage of immunization dropped from 86% in 2019 to 81% in 2021 (4).

In 2021, 25 million children were zero-dose (missing Penta-1) or under-vaccinated (missing Penta-3) (5). In 2019, 19.7 million children worldwide did not receive the third dose of vaccination against diphtheria, tetanus, and pertussis in their first year of life, a crucial sign of the effectiveness of immunization programs. Every year, 4.4 million children in sub-Saharan Africa die from communicable diseases that could be prevented by vaccination (6).

The Expanded Programme for Immunization (EPI) of Ethiopia is currently administering 11 different antigens to infants born in the country each year. The vaccination service is mainly provided in health centers and health posts through a combination of static, outreach, and mobile activities. Polio, measles, and meningitis vaccines are also being administered by large-scale periodic campaigns (7).

A study conducted in pastoral and semi-pastoral regions of Ethiopia revealed that 20.5% of children who received at least one vaccine did not take at least one of the subsequent doses. The dropout rate between pentavalent 1 and measles vaccinations was 14.1% followed by BCG and measles (10.1%), both of which are above 10%, the WHO maximum tolerable dropout rate (8).

Vaccination dropout refers to the proportion of children who did not get the subsequent vaccine among those who received the first vaccine. It is determined by calculating the proportion of children who dropped out from the immunization system between Penta-1 and Penta-3 or Penta-1 and MCV-1 (9, 10) or BCG and MCV-1, BCG and MCV-2, BCG and Penta-1, or BCG and Penta-3. Dropout rate can also be determined by using BCG as the entry vaccine and measles as the exit vaccine (proportion of children who did not get measles antigen among those who received BCG (10). Vaccination dropout rates are utilized to measure the continuity of the immunization program for children. According to the WHO, a dropout rate of >10% is unfavorable and indicates that a health facility has limitations in utilization. In contrast, a low dropout rate is indicative of good utilization and therefore of good service quality (11–13).

Gavi’s 5.0 Strategy is guided by a “missed communities, first priority” principle to prioritize children missing vaccinations, particularly those found in displaced and vulnerable communities. Almost half of these missed children reside in fragile and conflict-affected settings. Studies focused on immunization, including dropout, have not been extensively conducted in underserved settings including pastoralist regions, developing regions, newly established regions, conflict affected areas, underserved urban populations, internally displaced populations (IDPs), and refugee populations, especially after the two great challenges (COVID-19 and internal conflicts) Ethiopia has recently experienced. Hence, this study was conducted to estimate the prevalence of vaccination dropout, identify associated factors and explain the socioeconomic inequality in children aged 12–35 months in remote and underserved areas of Ethiopia.

## 2. Methods

### 2.1. Study design and settings

This study was part of a cross-sectional national evaluation survey that was conducted from May to July 2022. Prior to the quantitative survey, zero-dose and under-immunized areas were identified through qualitative situational and geospatial analyses based on secondary data sources. The study was implemented following a single round, cross-sectional survey design.

Considering that almost half of zero-dose and under-immunized children in low-income countries are located in hard-to-reach communities, conflict-affected settings, or disadvantaged urban areas (14), the study focused on populations in the following eight partly overlapping settings.

1. Pastoralist regions and populations: Afar and Somali regions, and specific pastoralist or semi-pastoralist settings in Oromia, Southern Nations, Nationalities, and Peoples (SNNP), Southwest Ethiopia Peoples, and Gambella regions.
2. Developing regions: Afar, Somali, Gambella, and Benishangul Gumuz regions.
3. Newly established regions: Sidama and Southwest Ethiopia Peoples regions.
4. Conflict affected areas: Selected settings in Afar, Amhara, Oromia, and Benishangul Gumuz regions.
5. Underserved urban populations: Urban slums in six selected cities (Addis Ababa, Bahirdar, Hawassa, Dire Dawa, Harar, and Adama) and rural areas under Dire Dawa City administration and Harari region.
6. Hard-to-reach areas in major regions: Selected remote districts in Amhara, Oromia, and SNNP regions.
7. IDPs: Selected IDP centers in Afar, Amhara, Oromia, and Benishangul Gumuz regions.
8. Refugees: Refugees from selected camps found in Somali, Afar, and Gambella regions (15).

### 2.2. Study participants

The target population were children under five years of age who lived in underserved, remote, and conflict-affected areas of Ethiopia. Children between the ages of 12 and 35 months were included in the study.

### 2.3. Sample size determination

The sampling design of the study was adopted from WHO’s 2018 immunization coverage cluster survey manual (16). The Cochran’s Single Proportion Sample Size Formula was employed to calculate the appropriate sample size for each target population (17) assuming 95% confidence level, 4% margin of error, 16% prevalence of zero-dose children (18), and 10% compensation for possible non-response. The following sample size formula was used to calculate the number of children required in a prevalence study: $n=\frac{Z2p(1-p)}{d2}$ where *n* is the total sample size needed, Z is the statistic corresponding to 95% confidence level which is 1.96, *P* is the prevalence of zero-dose children in previous studies in Ethiopia, and d is precision (corresponding to effect size) (19). Thus, it was necessary to have a sample size of 360 for each target population domains.

The Ethiopian DHS 2016 and Mini DHS 2019 data indicate that each enumeration area (EA) has an average of 12 children aged 12–35 months (18). Therefore, a minimum of 30 EAs were required for each population domain to recruit 360 children in each target population within 12–35 months, assuming that all children in the EA would be eligible for inclusion into the study. In urban slums, 40 EAs were randomly selected and 480 children were drawn (15).

The original plan was to include 4,080 children from 340 EAs (at least 360 samples per population domain) in the survey. Due to security concerns in certain study districts, 3,646 children aged 12–35 months from 340 EAs were enrolled in the actual survey. The total sample size was sufficient to enable subgroup analysis based on sex, age and other relevant background characteristics, including socioeconomic status (15).

### 2.4. Sampling procedure

A two-step procedure was employed to select children aged 12–35 months using a cluster sampling approach. The first step was to randomly select EAs from the total EAs available in each target population domain (15). The sampling frame used was the EAs delineated by the Central Statistical Agency (18) of Ethiopia for the recent census. In the case of urban slums, hotspot urban slums in Addis Ababa, Adama, Bahir Dar, Hawassa, Harar, and Dire Dawa cities were located, delineated, and EA maps were drawn by experienced cartographers. In the case of IDP and refugee camps, villages or clusters were considered as EAs. The second step was to list all eligible children in each EA and then select 12 children using a smartphone-based random number generator (15).

### 2.5. Data collection procedures and data quality assurance

Data were collected using pretested tools prepared in Amharic, Afan Oromo, Somali, Afar, and Sidama languages. Survey data were collected by 48 experienced enumerators and 24 supervisors using the CommCare digital app, an application system that is open-source, user-friendly, and compatible with major data analytics and visualization software. The CommCare app was utilized to gather information about individual children and households to guarantee high-quality data collection, cleaning, and monitoring in real time (20).

The recruitment process for enumerators and supervisors was based on their educational status (at least diploma holders in a health-related discipline), past experience in similar national surveys, and familiarity with the CommCare digital app (20). The enumerators and supervisors received a 5-day training before deployment that was guided by a structured training manual. The training covered the sampling approach, basic data collection principles, a line-by-line discussion of the questionnaire, an introduction to utilizing the CommCare digital app, mock interviews, field exercises, and an overview of basic ethical research practices that involves human subjects (15).

Data collectors were permitted to collect data from as many as six individuals per person per day. The quality of the data was verified by re-interviewing a third of all the study participants by the supervisors. Throughout the survey implementation period, the research team was closely monitoring the uploaded data (15).

### 2.6. Ascertainment of childhood vaccination

Three sources of information, including caregiver’s report, home-based (vaccination cards) reports, and facility-based reports, were used to determine the vaccination status of children, as recommended by the WHO. The child’s immunization status was determined by the review of the vaccination card when the mother or caregiver presented it. In the absence of an immunization card, the immunization status was assessed based on the self-reports/recalls of mothers/caregivers. Previous studies have validated the reliability of this method in ascertaining childhood vaccination in resource-limited settings with inadequate documentation of childhood immunization (20, 21).

### 2.7. Variables of the study

The primary outcome of interest of the study was vaccination dropout which is the proportion of children who did not get the subsequent vaccine among those who received the first vaccine.

The predictor variables included wealth index, marital status, child age, respondent age, time to walk to the health facility (one-way), maternal educational status, paternal educational status, caregiver’s employment status, antenatal care visits, postnatal care (PNC) services, place of residence, number of under-five children, skilled birth attendance (SBA), availability of health facility in the kebele (a small administrative unit), gender empowerment, child sex, and sex of household head.

Gender empowerment is a composite index measuring gender inequality in economic participation, decision-making mainly on health-related matters and power over economic resources.

The wealth index, which is a composite variable, measures the woman’s household living standards. As per the DHS program’s recommendation, it was calculated as a composite index of living standards. The wealth index was created based on the ownership of valuable assets and livestock, the land size for agriculture and housing purposes, materials used to construct homes, availability and access to basic social services such as electricity, banking, improved water sources, as well as methods for disposing of body waste. By using Principal Component Analysis, a total of 41 variables were reduced to nine factors. The components were combined into a score and then split into five quintiles (poorest, poorer, middle, richer, and richest) (15, 18).

### 2.8. Data management and statistical analysis

Data was collected using the CommCare digital app (22) and stored in a local server on a daily basis. It was exported to STATA version 17.0 (23) for advanced statistical analysis. The weighted and unweighted sample size was balanced by linearizing post-stratification weights.

Vaccination dropout was assessed using BCG as the entry vaccine and measles/Pentavalent as the exit vaccine doses. Accordingly, four dropout measures including BCG to Penta-1, BCG to Penta-3, BCG to MCV-1, and BCG to MCV-2 were developed. Additionally, two vaccination dropout measures that included Penta-1 to Penta-3 and Penta-1 to MCV-1 were computed using Penta-1 as the entry vaccine and Penta-3/or MCV-1 as the exit vaccine doses. Vaccination dropout was calculated as the proportion of children who did not get the subsequent vaccine among those who received the first vaccine.

In the immunization program, the first and third doses of Pentavalent vaccine are considered as tracer indicators (24). In the present study, the statistical analyses were performed using the Penta-1 to Penta-3 vaccination dropout estimate as an outcome variable i.e., the proportion of children who did not receive Penta-3 among those who received Penta-1.

The outcome variable is whether a child had dropped out of the third dose of Pentavalent vaccine. Predictor variables considered in the current study were selected based on proven relationships and predicting factors from published literatures and their biologically plausible association with vaccination dropout. These included wealth status, marital status, child age, respondent age, time to walk to health facility (one-way), maternal educational status, paternal educational status, caregiver’s employment status, ANC visits, PNC services, place of residence, number of under-five children, SBA, availability of health facility in the kebele, gender empowerment, child sex, and sex of household head. A univariable generalized estimating equation analysis was carried out to examine the relationship between the outcome variable and selected predictor variables. Then, all potential predictors with a *p*-value less than 0.05 in the univariable analysis were fitted into the final generalized estimating equation model. Outputs were summarized using crude odds ratio (23) and adjusted odds ratio (AOR) with 95% confidence interval (25). A *p*-value of ≤ 0.05 was considered statistically significant.

#### 2.8.1. Vaccination dropout inequality by wealth status

Vaccination dropout inequality by wealth status was estimated using the Concentration Curve and Concentration Index (CCI). The concentration curve depicts the cumulative percentage of the population, ranked by wealth status, from the poorest to the richest on the x-axis against the cumulative percentage of the dropout status of children on the y-axis. The curve would be aligned with the 45 degrees line if all children had an equal percentage of dropouts regardless of their wealth status, which indicates the presence of equality in vaccination dropout. The concentration curve falling below the 45 degrees line of equality implies that dropout is more concentrated among the rich. The opposite is true if the curve falls above the line of equality. The concentration index is a measure of two times the area between the line of equality and the concentration curve. The index is determined by a value between −1 and +1; an index of 0 shows the presence of uniform vaccination dropout between the poor and the rich. Wealth-related inequalities can be observed in one of the two forms. The first is when there is an uneven concentration of dropout among the rich, the concentration index takes on a positive value. The second is a concentration index taking a negative value that implies a high concentration of vaccination dropout status among the poor (26).

The CI can then be measured as follows: twice the covariance of the health variable and the ranking of the living standards variable r all divided by the mean of the health measure (μ). Greater absolute CI values indicate greater inequality in vaccination dropout.

$$CI=\frac{2}{\mu }\mathrm{cov}\;(h,r)$$

This study utilizes the Erreygers corrected concentration index that is algebraically expressed as shown below.

$$E(h)=\frac{4\mu }{b-a}CI$$

Where μ is the mean of the health variable (vaccination dropout), *CI* is the standard CI, with b and a representing the upper and lower bounds of the outcome variable (h). In our study, the range b–a is unity, as the outcome variable is binary (27).

## 3. Results

### 3.1. Sociodemographic characteristics

A total of 3,646 mothers/caregivers with children aged 12–35 months participated in the study with a response rate of 97.7%. Over half of (54%) respondents were between 25 and 34 years of age and the majority of them (59.2%) had no formal education. More than 81% of respondents were from rural areas and 17% of them were from Afar region. Nearly 91% of the respondents were married/living together and 57% were unemployed at the time of the survey (Table 1).

**Table 1:** Socio-demographic characteristics of respondents and children in underserved settings of Ethiopia, 2022.

Characteristics	Frequency	Percent
Child’s sex		
Boy	1,985	54.4
Girl	1,661	45.6
Child’s age (months)		
12–23	1,849	50.7
24–35	1,797	49.3
Caregiver’s age		
15–24	875	24.0
25–34	1,969	54.0
35–44	572	15.7
45 or above	105	2.9
Do not know	126	3.5
Caregiver’s educational status		
No formal education or preschool	2,158	59.2
Primary education	788	21.6
Secondary education	616	16.9
Tertiary education	84	2.3
Marital status		
Not ever married	43	1.2
Married/Living together	3,312	90.8
Separated	83	2.3
Divorced	110	3.0
Widowed	98	2.7
Place of residence		
Urban	677	18.6
Rural	2,969	81.4
Caregiver’s employment status		
Unemployed	2,098	57.6
Employed	1,548	42.4
Region^a^		
Afar	636	17.4
Amhara	372	10.2
Oromia	431	11.8
Somali	480	13.2
Benishangul Gumuz	216	5.9
Southern Nations Nationalities and peoples	300	8.2
Sidama	239	6.6
Southwest Ethiopia	181	5.0
Gambella	479	13.1
Harari	60	1.6
Addis Ababa	192	5.3
Dire Dawa	60	1.6
Household size		
2–5	2,044	56.0
6 or above	1,602	44.0

^a^ Unweighted sample size.

### 3.2. Childhood immunization

Of the 3,646 children, nearly 71% had received BCG. The proportion of children who had received the first dose of Pentavalent vaccine was 68% and 39% of them had received the third dose of the pentavalent vaccine. Table 2 below shows the number of children who had received various antigens against common childhood illnesses according to vaccination cards, medical records, or caregivers’ self-reports.

**Table 2:** Number/proportion of children who had received various antigens according to the routine immunization schedule of Ethiopia, 2022.

Type of antigen	Number of children who had received vaccines	Percentage (%)
BCG	2,581	70.8
Penta-1	2,464	67.6
Penta-3	1,410	38.7
MCV-1	2,442	67.0
MCV-2	1,271	34.9

### 3.3. Vaccination dropout estimates in remote and underserved settings of Ethiopia

**Figure 1:**
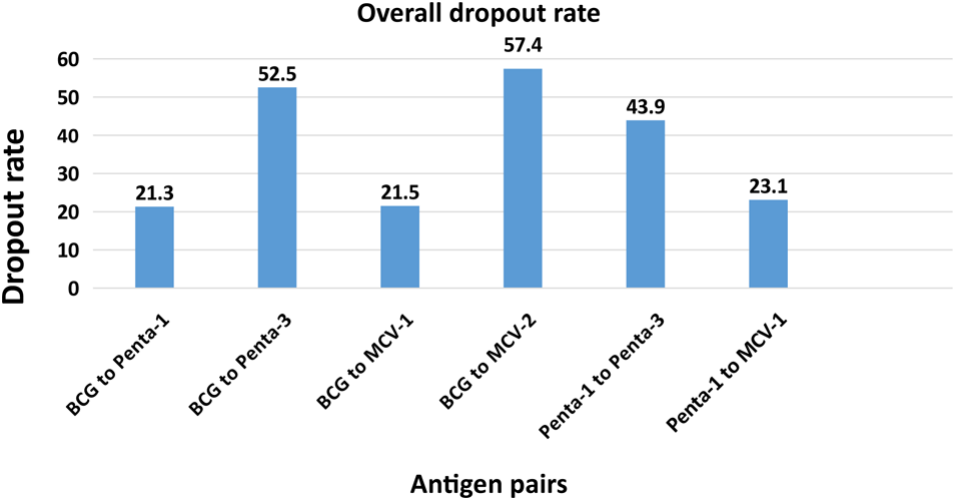
Percentage of vaccine dropout according to various measures by pair of antigens, 2022.

Vaccination dropout estimates varied across the different pairs of antigens, target populations, and the type of measurement methods used. Overall, the highest vaccination dropout estimate was 57.4% based on BCG and MCV-2 vaccines, whereas the lowest dropout was 21.3% for BCG to Penta-1 pairs (Figure 1). When pentavalent-1 was considered as an entry vaccine and Pentavalent-3 as an exit vaccine, the overall dropout became 44%. Newly formed regions had the highest vaccination dropout estimate of 73% based on BCG to MCV-2 measure, followed by 70% in IDP camps according to the BCG to pentavalent-3 measure. Urban slums had the lowest dropout estimates across all measures (Table 3).

**Table 3:** Vaccination dropout estimates according to the different types of measures & target populations in remote & underserved settings of Ethiopia, 2022.

Target population group	Vaccination dropout according to various measures					
** **	BCG to Penta-1, *n* (%)	BCG to Penta-3, *n* (%)	BCG to MCV-1, *n* (%)	BCG to MCV-2, *n* (%)	Penta-1 to Penta-3, *n* (%)	Penta-1 to MCV-1, *n* (%)
Urban slums	20 (4.9)	55 (13.6)	44 (11.0)	171 (42.8)	46 (11.2)	41 (10.0)
Conflict affected regions	52 (24.3)	109 (51.6)	41 (19.2)	105 (49.4)	78 (38.6)	51 (25.4)
IDPs	81 (38.0)	148 (70.0)	50 (23.6)	127 (60.0)	88 (57.1)	27 (17.6)
Hard-to-reach in agrarian regions	89 (16.0)	260 (46.5)	105 (18.8)	306 (54.7)	232 (40.0)	125 (21.5)
Pastoralist population	122 (15.3)	412 (52.8)	142 (17.9)	467 (58.8)	453 (49.3)	215 (23.4)
Developing regions	179 (27.5)	449 (68.8)	130 (20.0)	394 (60.4)	336 (60.1)	158 (28.2)
Newly established regions	76 (29.9)	144 (56.7)	99 (39.0)	186 (73.1)	111 (48.8)	82 (36.0)
Refugees	50 (19.9)	139 (55.4)	83 (33.1)	174 (69.3)	99 (45.4)	73 (33.5)
All^a^	533 (21.3)	1,317 (52.5)	540 (21.5)	1,438 (57.4)	1,063 (43.9)	558 (23.1)

^a^ The sum of the target population groups exceeds the total as some populations contributed to more than one domain.

### 3.4. Determinants of vaccination dropout rate in remote and underserved settings of Ethiopia

Independent variables that were found statistically significant in the generalized estimating equation analysis at the *p*-value of ≤ 0.05 included the following: children born from mothers/caregivers who were at the lower level of the wealth quintile, caregivers who were older than 45 years, caregivers who did not have any formal education, caregivers who were working outside of their homes, caregivers who did not attend the minimum recommended number of ANC contacts (at least four times), postnatal contacts, and caregivers who did not attend a SBA during pregnancy were found to have higher dropout rates compared to their counterparts. Dropout rates were also higher for children from mothers/caregivers who were living in areas where a health facility was located very far from their homes (30 min walking distance or more). Low gender empowerment and a household with three or more under-five children were also contributing factors for high vaccination dropout (Table 4).

**Table 4:** Generalized estimating equation model showing predictors of vaccine dropout among children aged 12–35 months in remote and underserved settings of Ethiopia, 2022.

Covariates (*n* = 2,417)	Dropout, *n* (%)	COR (95% CI)	AOR (95% CI)^a^
Wealth status			
Poorest	243 (59.0)	2.55 (1.82–3.58)*	1.87 (1.38–2.52)*
Poorer	252 (47.5)	1.67 (1.24–2.25)*	1.15 (0.62–2.12)
Middle	236 (44.0)	1.45 (1.16–1.81)*	1.06 (0.77–1.44)
Richer	208 (36.0)	1.04 (0.69–1.57)	0.92 (0.61–1.40)
Richest	145 (35.0)	1	1
Maternal educational status			
No formal education	699 (49.9)	1.54 (1.42–1.68)*	1.31 (1.11–1.53)*
Primary education	212 (39.1)	1	1
Secondary education	155 (34.6)	0.84 (0.53–1.33)	1.25 (0.86–1.82)
Tertiary education	18 (23.6)	0.47 (0.16–1.36)	1.04 (0.41–2.59)
Caregiver’s employment status			
Working	513 (49.4)	1.52 (1.09–2.12)*	3.67 (1.24–10.86)*
Not working	570 (40)	1	1
Paternal educational status			
No formal education	538 (49.9)	1.42 (1.22–1.65)*	1.30 (1.01–1.67)*
Primary education	196 (40.7)	1	1
Secondary education	199 (38.3)	0.89 (0.71–1.13)	0.92 (0.69–1.23)
Tertiary education	45 (25.3)	0.46 (0.17–1.28)	0.61 (0.25–1.51)
Place of residence			
Urban	219 (42.6)	0.95 (0.64–1.43)	1.3 (0.96–1.78)
Rural	865 (44.3)	1	1
Child age			
12–23 months	508 (40.2)	1	1
24–35 months	575 (48.0)	1.37 (1.14–1.65)*	1.41 (1.11–1.79)*
Number of under-five children			
One	441 (36.2)	1	1
Two	557 (51.0)	1.81 (1.43–2.27)*	1.77 (1.45–2.16)*
Three or more	85 (55.4)	2.02 (0.78- 5.21)	1.89 (1.00–3.64)*
Marital status			
Not married	100 (50.1)	1	1
Married	984 (43.4)	0.76 (0.40–1.45)	1.15 (0.69–1.9)
Caregiver’s age			
15–24 years	224 (38.5)	1	1
25–34 years	642 (46.8)	1.41 (1.11–1.79)*	1.27 (1.15–1.39)*
35–44 years	176 (46.9)	1.39 (1.05–1.85)*	1.35 (0.96–1.90)
45 years or above	24 (42.2)	1.16 (0.75–1.78)	12.49 (3.87–40.33)*
ANC visits			
Less than 4 contacts	637 (48.9)	1.37 (1.00–1.96)*	1.17 (1.00–1.82)*
4 or more contacts	409 (39.7)	1	1
SBA			
No	455 (52.6)	1.61 (1.23–2.08)*	1.64 (1.21–2.27)*
Yes	592 (40.3)	1	1
PNC			
No	588 (50.0)	1.57 (1.05–2.35)*	1.66 (1.15–2.39)*
Yes	459 (39.6)	1	1
Availability of health facility in the kebele			
Yes	1015 (43.1)	1	1
No	69 (61.8)	2.07 (1.13–3.80)*	1.49 (0.97–2.27)
One-way walking distance to the nearest health facility			
30 min or less	481 (39.3)	1	1
30 min or above	603 (48.6)	1.45 (1.16–1.82)*	1.33 (1.22–1.45)*
Gender empowerment			
Low	160 (60.1)	2.21 (1.68–2.93)*	1.63 (1.24–2.15)*
Medium	120 (43.3)	1.11 (0.93–1.31)	1.08 (0.97–1.21)
High	704 (40.9)	1	1
Child sex			
Boy	607 (45)	1.08 (0.89–1.29)	1.07 (0.84–1.36)
Girl	476 (42.8)	1	1
Sex of household head			
Female	120 (56.2)	1.74 (0.90–3.35)	1.88 (0.66–5.33)
Male	964 (42.8)	1	1

^a^ adjusted for all variables presented in the table.

^*^ Significant association at 5% level of significance.

Children born from mothers/caregivers who were at the lower level of the wealth quintile had the highest dropout rates compared to their counterparts [AOR (95%CI) = 1.87 (1.38–2.52)]. Children of parents who reported not having any formal education had higher levels of dropout compared with children whose mothers/caregivers and fathers had some years of schooling [AOR (95%CI) = 1.31 (1.11–1.53)] and [AOR (95%CI) = 1.30 (1.01–1.67)], respectively.

The odds of vaccine dropout rate among children who were born from mothers/caregivers who were working at the time of the survey was nearly four times [AOR (95%CI) = 3.67 (1.24–10.86)] compared to those children who were born from mothers/caregivers who were not working. Children who were born from mothers/caregivers who had no PNC follow-up contacts were nearly two times more likely to drop out of vaccination compared to those children who were born from mothers/caregivers who had attended a PNC follow-up visit [AOR (95%CI) = 1.66 (1.15–2.39)]. In addition, the odds of childhood vaccination dropout among children who were born from mothers/caregivers who had less than four ANC contacts had a higher likelihood of dropping out of vaccination [AOR (95%CI) = 1.17 (1.00–1.82)] (Table 4).

Children who were born from mothers/caregivers who received a service from a SBA during pregnancy were less likely to drop out of vaccination compared to those children who did not get the service [AOR (95%CI) = 0.61 (0.44–0.83)]. The likelihood of vaccine dropout was twelve times higher among children who were born from mothers/caregivers that were older than 45 years at the time of the survey compared to those children who were born from mothers/caregivers that were younger than 45 years [AOR (95% CI) = 12.49 (3.87–40.33)]. The closer the mothers’/caregivers’ home to the nearest health facility was also found to reduce vaccination dropout rate [AOR (95% CI) = 1.33 (1.22–1.45)]. Children of mothers who were less empowered were more likely to drop out of vaccination compared to those children of mothers who were well empowered [AOR (95%CI) = 1.63 (1.24–2.15)] (Table 4).

### 3.5. Vaccination dropout inequality by wealth status

**Figure 2:**
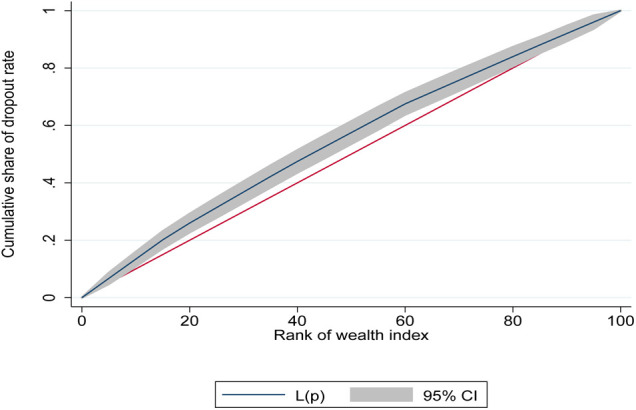
Concentration curve for vaccination dropout among children aged 12–35 months in remote and underserved settings of Ethiopia, 2022.

Inequalities in vaccination dropout were significantly and disproportionately concentrated among the poor (CI = −0.17865141; *p* < 0.01). The negative value CI for children who dropped out of vaccination confirms pro-poor distributions (Figure 2). It was also evident from this study that Addis Ababa city administration had the highest and Gambella region had the lowest vaccination dropout inequalities by wealth (Table 5).

**Table 5:** Vaccination dropout inequality by wealth status in regions, 2022.

Region	*N*	Mean	Margin
Afar	355	−0.2934411	1
Amhara	288	−0.118632	0.0570416
Oromia	309	−0.2033019	0.0152045
Somali	216	−0.2034953	0.2036885
Benishangul Gumuz	153	−0.2794761	0.0040176
SNNPR	239	−0.1845429	0.0989508
Sidama	141	0.0930139	0.0843412
Southwest	121	−0.1628358	0.1206579
Gambella	366	−0.03338	0.2458167
Harari	43	−0.4165674	−0.1330737
Addis Ababa	181	−0.869686	−0.5861923
Diredawa	52	−0.245111	0.0383828
Total	2,464	−.1772053	–

## 4. Discussion

This cross-sectional national evaluation survey aimed at estimating the prevalence of vaccine dropout rate and its associated factors among children aged 12–35 months in remote and underserved areas of Ethiopia. It is the first extensive national study on immunization after the two great challenges (COVID-19 and internal conflicts) Ethiopia has recently experienced.

In the immunization program, the first and third doses of Pentavalent vaccine are referred to as tracer indicators (1, 24). In this study, apart from the Pentavalent vaccine, other antigens were also used to estimate vaccination dropout. Overall dropout as well as dropout disaggregated by different population domains were performed. In addition, contributing factors for vaccination dropout and inequality of dropout based on wealth status were also explored using generalized estimating equation and concentration curve/index, respectively.

The magnitude of vaccination dropout ranged from 21.3% based on BCG to Penta-1 to 57.4% based on BCG to MCV-2. Using Penta-1 to Penta-3 measures, vaccination dropout was 44%. All the dropout estimates in our study were higher compared to the maximum tolerable dropout level of 10% by the WHO. This might be indicative of poor performance of immunization programs in underserved settings of Ethiopia. Vaccination dropout estimates of the present study in the pastoralist population of Ethiopia were higher compared to dropout estimates from a previous study conducted in the same study settings in Ethiopia. In this study, dropout rates using penta-1 to Penta-3 was 49.3% and Penta-1 to MCV-1 was 23.4% which are much higher than dropout estimates of a previous study done in the same study setting using same pairs of antigens, which found rates of 22.1% and 9.0%, respectively (8). In addition, dropout estimates of the present study were higher compared to a dropout estimate of another study (33.1%; Penta-1 to Penta-3) conducted in Jigjiga district, Somali regional state of Ethiopia (28). The dropout rate in this study (44%) was also higher than 15% as reported in the mini demographic and health survey 2019 (Penta-1 to Penta-3) (29). The higher dropout rates in the present study might be attributed to the COVID-19 pandemic and internal conflicts.

Globally, childhood vaccination is influenced by parental education (30, 31). Our study has shown that children born from mothers/caregivers and fathers who reported not attending any formal education had higher levels of dropout compared with their counterparts and, respectively. This is consistent with studies conducted in Nepal, Kenya, Uganda, Guinea Bissau, Ethiopia, Nigeria, and Benin where children of uneducated mothers and fathers reported to have a higher vaccination dropout (32–38). Previous studies from rural India and rural Mozambique also reported that children whose mothers had no schooling were more likely to drop out of vaccination compared with children whose mothers had some years of schooling (39–41). Moreover, our study indicated that children of working mothers/caregivers had a higher likelihood of being dropped out from vaccination programs compared to their counterparts. This is also in line with a study done in Nepal. This might be due to the fact that working mothers have little time to stay with and care for their children (32).

Our study also showed the inverse relationship between wealth status and vaccination dropout. Children born from mothers/caregivers who were at the lower level of the wealth quintile had the highest dropout compared to their counterparts. This is supported by studies conducted in Pakistan (42), Kenya (31), and Nigeria (37). However, a study from Nepal by Thapa et al. contradicts with our finding that stated that children from middle and rich families reported a higher dropout (32).

The 2016 ANC model of WHO recommends a minimum of eight ANC contacts throughout pregnancy (43). Our study showed that mothers who had fewer than four or no ANC visits and those who did not seek help from SBAs showed higher dropout rates than others. This might be because ANC contacts provide information about childbirth, newborn care, vaccinations, and the growth of children. In other words, inadequate ANC visits, late visits, or fewer than the recommended number of visits have been correlated with poor newborn health. This finding is supported by other studies conducted in Ethiopia, Nepal, Benin, and 69 low- and middle-income countries (13, 32, 38, 44).

Children who were born from mothers/caregivers who had no PNC follow up contacts were nearly two times more likely to drop out of vaccination compared to those children who were born from mothers/caregivers who had attended PNC follow-up visits. This finding aligns with a study conducted in North Gondar zone and Laelay Adiabo districts of Ethiopia and a study from Benin which concluded that the likelihood of defaulting from completion of child vaccination was higher among mothers/caregivers who did not attend PNC after birth as compared to those attended at least once (1, 38, 45). In addition, our results are also consistent with studies conducted in Kenya and Cameroon which showed that failure to attend PNC was a barrier to complete vaccination. The possible explanation of this might be missed opportunity to get advice on the benefits of child vaccination that ultimately leads mothers/caregivers to drop out of child vaccination (46, 47).

In our study, distance to the health facility was found to contribute to vaccination dropout. Closer distance from the mothers’/caregivers’ homes to a health facility correlated with lower vaccination dropout rates. This is consistent with findings from Nigeria, Ethiopia, Kenya, Cameroon, and Mozambique (13, 33, 47–51). The study from Kenya showed that residing over 5 km away from a health facility was associated with higher odds of dropping out. Lack of access to health facilities in the neighborhood was also found to contribute to high vaccination dropout. This aligns with results from a community-based participatory research study conducted in Mozambique which found that vaccination dropout was related to a lack of access to healthcare facilities (12, 40).

The present study has shown that the wealth status of mothers/caregivers contributed to vaccination dropout inequality by wealth status. Inequalities in vaccination dropout were disproportionately concentrated among the poor. The negative value (CCI = −0.17865141) for children who dropped out of vaccination confirms pro-poor distributions. Addis Ababa city administration and Gambella region have the highest and the lowest inequality of vaccination dropout by wealth, respectively. However, this does not mean that Addis Ababa city administration and Gambella have the highest and lowest dropout rates. Although vaccinations are provided free-of-charge in Ethiopia, there are indirect costs that mothers/caregivers are required to pay (for instance, transportation costs) which often limits the uptake of maternal and child health services, including vaccination. In this regard, mothers/caregivers at the lower level of the wealth category are more exposed to such challenges in accessing healthcare facilities, which ultimately leads to vaccination dropout. Our finding is consistent with studies conducted in Nigeria, Benin, and the Democratic Republic of the Congo. Using a nationally representative data, Ataguba et al. stated that existing immunization coverage disparities disproportionately benefited the rich (37, 38, 52, 53).

Maternal age was significantly associated with vaccination dropout in the present study. The likelihood of vaccine dropout was twelve times higher among children who were born from mothers/caregivers that were older than 45 years at the time of the survey compared to those children who were born from mothers/caregivers that were younger than 45 years. This is in line with a previous study conducted in Ethiopia (41). It was evident from this study that the odds of vaccination dropout in children from women with low levels of empowerment was 1.63 higher compared to children from women with high levels of empowerment. This aligns with studies conducted in Mozambique and the Democratic Republic of the Congo (51, 53).

This study had several strengths worth mentioning. Although study settings included areas where there were active conflicts, the study team still managed to collect data in those areas. Ascertainment of child vaccination dropout was based on information gathered from multiple sources. Vaccination cards, medical records, and maternal recall were used to ascertain vaccination dropout. This triangulation helped to verify and validate the results. The use of digital applications and experienced data collectors helped to ensure high-quality data was gathered. The results from this study could be used as a baseline to reduce vaccination dropout in underserved settings of Ethiopia.

The study also had some limitations, including difficulty to make a causal inference and susceptibility to biases such as nonresponse bias and recall bias which are directly linked to the study design i.e., cross-sectional. Mothers/caregivers who did not possess EPI cards might forget the vaccination status of their children and this ultimately might lead to misclassification.

## 5. Conclusion and recommendations

Vaccination dropout estimates were high among children residing in remote, underserved, and conflict affected settings of Ethiopia. Poor wealth quintile, uneducated parents, advanced maternal age, low women empowerment, inaccessibility of health facilities, and limited utilization of maternity care services were found to contribute to vaccination dropout.

Strengthening horizonal integration of immunization services with maternal & child health services such as antenatal and postnatal cares, sick childcare, nutritional screening, growth monitoring, oral rehydration therapy training sessions, vitamin A supplementation and family planning will reduce the number of children who are eligible for vaccination but do not receive one or more of the vaccine doses i.e., missed opportunities for vaccination. Empowering women is also another vehicle to reduce vaccination dropout. Strengthening outreach campaigns, supplementary immunization activities (SIAs), Periodic Intensification of Routine Immunization (PIRI) in remote and underserved areas will help reduce vaccination dropout. Policy makers in Ethiopia need to address the disproportionate concentration of vaccination dropout among the poor by strengthening women’s utilization of healthcare services and the accessibility of health facilities in rural kebeles.

## Data Availability

The raw data supporting the conclusions of this article will be made available by the authors, without undue reservation.
